# Update on North American tick-borne diseases and how to diagnose them

**DOI:** 10.1128/jcm.00807-23

**Published:** 2025-07-11

**Authors:** Kyle G. Rodino, Elitza S. Theel, Bobbi S. Pritt

**Affiliations:** 1 Department of Pathology and Laboratory Medicine, Perelman School of Medicine, University of Pennsylvania 14640 , Philadelphia, Pennsylvania, USA; 2 Department of Laboratory Medicine and Pathology, Mayo Clinic 195112 , Rochester, Minnesota, USA; Vanderbilt University Medical Center, Nashville, Tennessee, USA

**Keywords:** tick, tick-borne, vector, Lyme

## Abstract

Recent decades have seen a rise in the incidence of tick-borne diseases in the US, along with an increased number of pathogens transmitted by ticks, and geographic expansion of tick populations. A variety of laboratory testing methodologies are available for the diagnosis of tick-borne diseases, including serology, microscopy, and molecular-based methods. The preferred approach varies by the specific disease, locally available test options, and the stage of illness at patient presentation. This mini-review focuses on updates in our understanding of the epidemiology of tick-borne diseases in the US and advances in the field of laboratory diagnostics.

## INTRODUCTION

Tick-borne diseases (TBDs) pose a significant and growing health challenge in the United States (US). Ticks transmit more than 75% of all vector-borne disease (VBD) cases reported each year to the US Centers for Disease Control and Prevention (CDC), greatly outnumbering cases transmitted by mosquitoes, fleas, and other arthropods (1). The incidence of TBDs continues to rise, with reported cases more than doubling since 2004 (2). Actual case numbers are likely much greater due to underreporting, with Lyme disease (LD) cases estimated to be eight to 12 times higher than reported (3). The corrected estimate places LD as the third most common nationally notifiable infectious disease in the US in 2019 (4).

**Fig 1:**
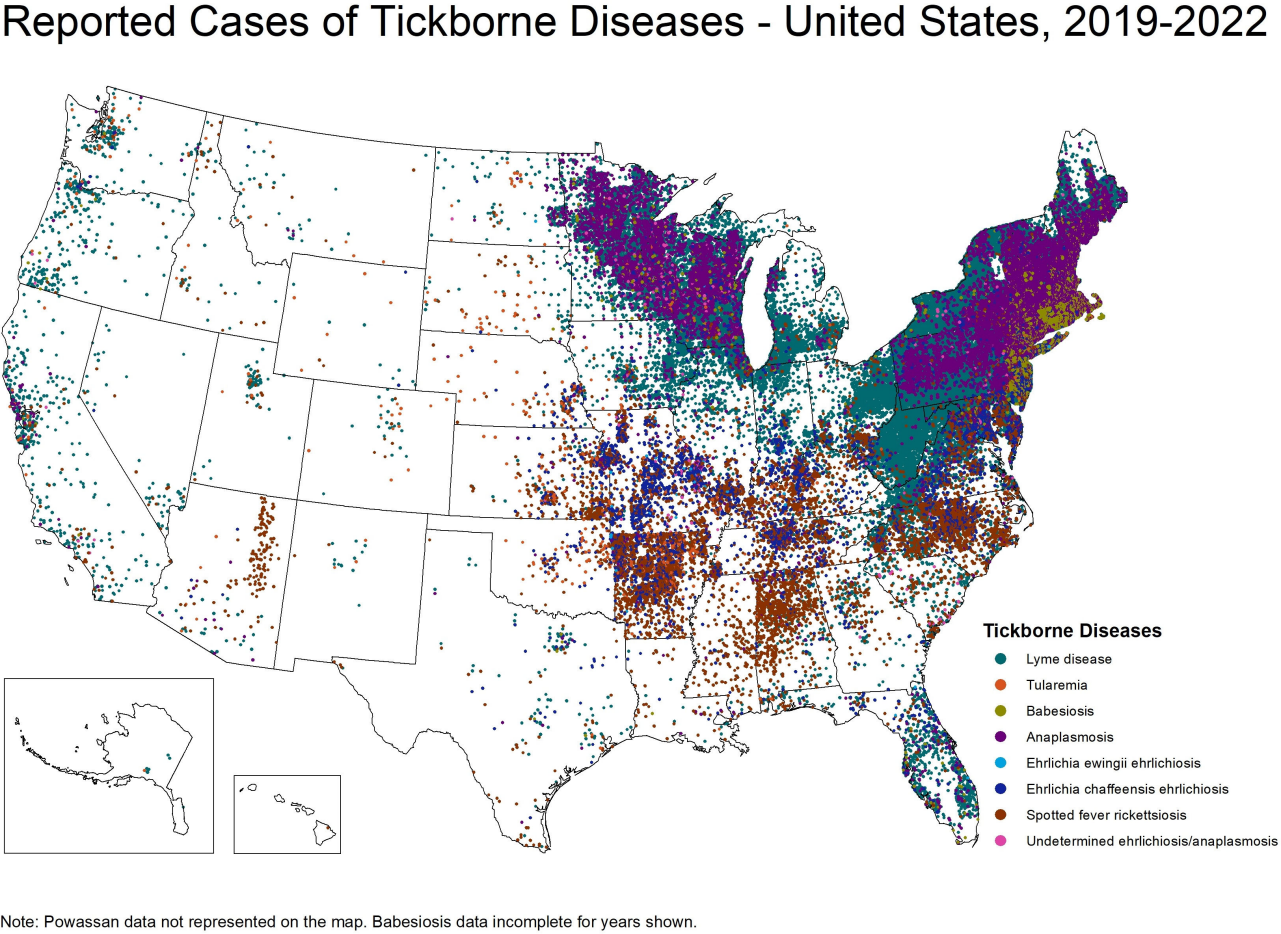
Centers for Disease Control and Prevention (CDC). Reported cases of tick-borne diseases in the US from 2019 to 2022.

In addition to increased cases of known TBDs, recent decades have seen an increased number of newly recognized tick-borne pathogens, particularly those transmitted by ixodid (hard) ticks, such as *Ixodes scapularis* and *Amblyomma americanum*. While LD, anaplasmosis, ehrlichiosis due to *Ehrlichia chaffeensis* and *E. ewingii*, babesiosis, Rocky Mountain spotted fever, tick-borne relapsing fever (RF), and tularemia remain the most common TBDs in the US, the past two decades have witnessed the emergence of five additional human bacterial pathogens (*Rickettsia parkeri* [5], *Rickettsia philippi* [364D] [6], *Ehrlichia muris eauclairensis* [7*]*, *Borrelia miyamotoi* [8], and *Borrelia mayonii* [9]) and three human viral pathogens (Heartland virus [10], Bourbon virus [11], and Lone Star virus [12]). Nucleic acid of “*Candidatus* Borrelia johnsonii” (13) and an *Anaplasma bovis-*like bacterium (14) have also been detected in human blood specimens submitted for TBD testing, although their role as human pathogens is currently unknown. Table 1 lists the common TBDs in the US, along with their etiologic agents, vectors, and geographic distribution. The distribution of the common TBDs in the US is shown in Fig. 1.

**TABLE 1:** Tick-borne diseases in the US with their etiologic agents, tick vectors, and endemic regions

Disease	Primary etiologic agent(s)	Primary vector(s)	Primary endemic region(s)^*c*^	# of reported cases in 2019^*d*^
Anaplasmosis	*Anaplasma phagocytophilum*	*Ixodes scapularis*, *I. pacificus*	Northeast, Upper Midwest	5,655
Babesiosis	*Babesia microti*	*I. scapularis*	Northeast, Upper Midwest	2,418
	*Babesia duncani*	*Dermacentor albipictus*	Pacific west	
	*Babesia divergens-like* organisms	Unknown	Central	
*Borrelia miyamotoi* disease (hard tick relapsing fever)	*Borrelia miyamotoi*	*Ixodes scapularis*, *I. pacificus*	Northeast, Upper Midwest	83 (15)
Bourbon virus disease	Bourbon virus (*Thogotovirus*)	*Amblyomma americanum*?	Midwest, south	N/A
Colorado tick fever	Colorado tick fever virus (*Coltivirus*)	*Dermacentor andersoni*	West	N/A
Ehrlichiosis	*Ehrlichia chaffeensis*	*A. americanum*	Southeast, South Central	2,093
	*Ehrlichia ewingii*	*A. americanum*	Southeast, South Central	
	*Ehrlichia muris eauclairensis*	*I. scapularis*	Upper Midwest	
Heartland virus disease	Heartland virus (*Bandavirus*)	*A. americanum*	Central	N/A; >60 as of 2022
Lyme disease^*b*^	*Borrelia burgdorferi*	*I. scapularis*, *I. pacificus*	Northeast, Upper Midwest	34,945 (old-reporting definition)
	*Borrelia mayonii*	*I. scapularis*	Upper Midwest	
Pacific Coast tick fever	*Rickettsia philipii* (type strain “364D”)	*Dermacentor occidentalis*	Pacific West	Included in total of spotted fever rickettsioses (below)
Powassan virus infection	Powassan virus (flavivirus), lineages I and II (deer tick virus)	*Ixodes cookei* (lineage) I), *I. scapularis* (lineage II)	Northeast, Upper Midwest	43
Relapsing fever borreliosis (soft tick)	*Borrelia hermsii*, *B. turicatae*	*Ornithodoros* spp.	West, Central	N/A; 251 cases from 2012 to 2021 (16)
Spotted fever rickettsioses^*a*^	*Rickettsia rickettsii*	*Dermacentor variabilis*, *D. andersoni*, *Rhipicephalus sanguineus*	Southeast, Central, Tribal Southwest	5,207
	*Rickettsia parkeri*	*Amblyomma maculatum*	Southeast	
Tularemia	*Francisella tularensis*	*A. americanum*, *D. variabilis*, *D. andersoni*		274

^*a*^ Rarely, other tick-borne *Rickettsia* species have been implicated in human disease in North America (17).

^*b*^ *Borrelia bissetti* is a member of the *Borrelia burgdorferi sensu latu* complex that has occasionally been detected in humans, but its significance as a pathogen is unclear (18).

^*c*^ TBDs may be found outside of these primary endemic regions.

^*d*^ Data from the US Centers for Disease Control and Prevention unless otherwise noted. Data are not available (N/A) for some tick-borne diseases.

**Fig 2:**
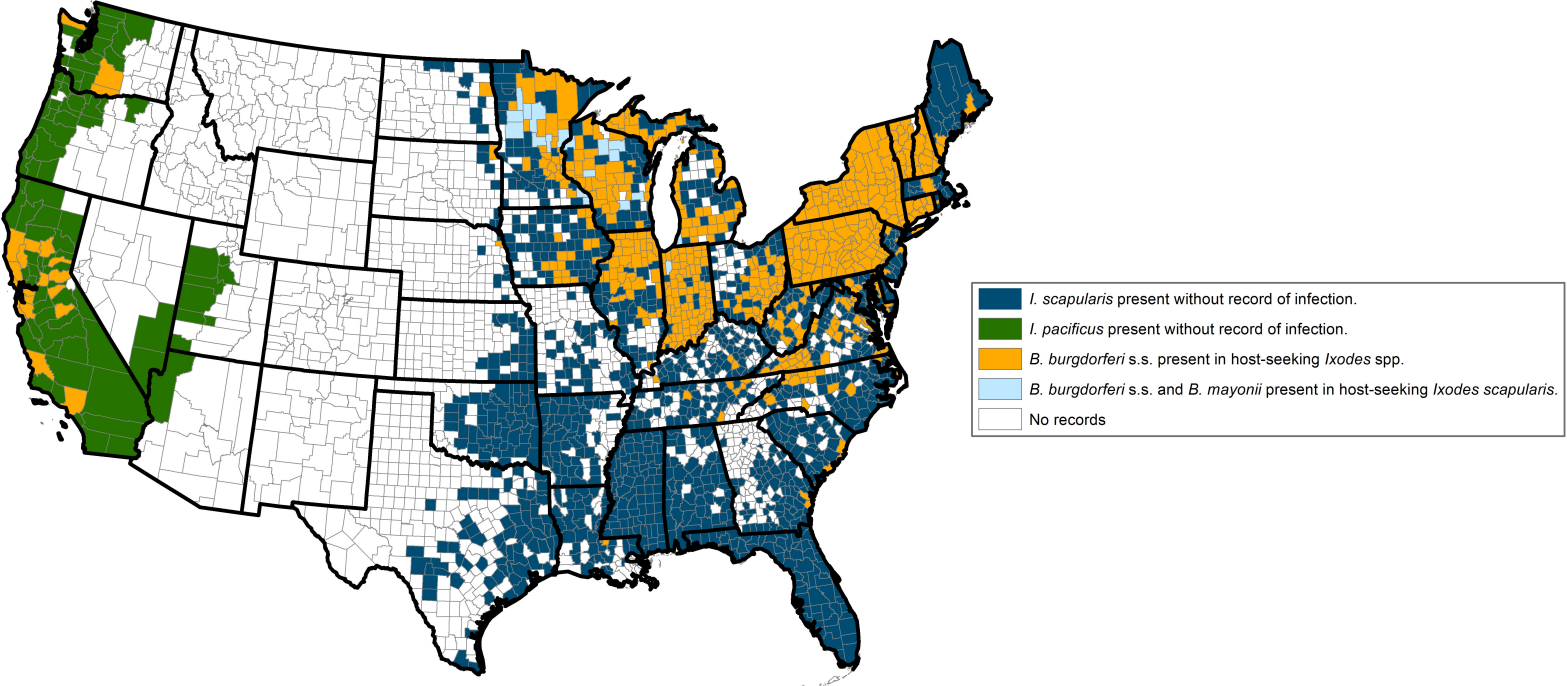
Centers for Disease Control and Prevention (CDC). Reported county-level distribution of *Borrelia burgdorferi* and *B. mayonii* in host-seeking *Ixodes scapularis* (eastern and midwestern states) or *I. pacificus* (western states) relative to the previously reported distribution of these ticks. Ticks were considered present in a county if one or more ticks were recorded. Counties where ticks had been reported without detection of *Borrelia* spp. may indicate either that the pathogen was not detected in tested samples or that testing of the ticks was not performed. Similarly, counties classified as “no records” could arise from a lack of either tick sampling efforts or from a lack of reporting or publishing the results of sampling efforts.

Finally, there has been a notable expansion of tick populations across the nation, including populations of the two most important disease vectors in the US, *I. scapularis* and *A. americanum* (19). *I. scapularis*, commonly known as the “black-legged” or “deer” tick, vectors the greatest number of TBDs in North America (Table 1) and has substantially increased its range during the 20th century following changes in land use and expansion of white-tailed deer populations (*Odocoileus virginianus*) (19). Today, *I. scapularis* is found throughout the Northeast, most of the Southeast, and significant regions of the Midwest (Fig. 2). In 2016, Eisen et al. (20) demonstrated that *I. scapularis* had been documented in 45.7% of the 3,110 counties in the continental US, while the other Lyme disease vector, *Ixodes pacificus*, has been documented in 3.6%. Combined, these ticks are present in nearly half of all counties in the continental US, reflecting a 44.7% increase in the number of counties harboring Lyme disease vectors compared with a map published in 1998 (20, 21).

*A. americanum*, also known as the lone star tick (named for the distinctive white spot on the dorsum of adult females), has also been expanding rapidly beyond the American South where it was originally described, moving into areas across the northern and midwestern states (19). Populations are now established throughout much of New England and extend as far north into the Midwest as Michigan and Wisconsin and as far west as Nebraska and South Dakota. A 2019 study (22) using ecological niche modeling predicted that further westward and northward expansion of *A. americanum* is to be expected with the ongoing climate change. Unlike the stationary questing behavior of *I. scapularis*, *A. americanum* is an aggressive biter that will actively pursue its hosts across many meters. It is the vector for ehrlichiosis due to *E. chaffeensis* and *E. ewingii*, tularemia due to *Francisella tularensis*, Heartland virus disease, and Bourbon virus disease (Table 1). It is also the primary tick implicated in southern tick-associated rash illness (STARI) and alpha-gal syndrome (a.k.a. meat allergy).

**Fig 3:**
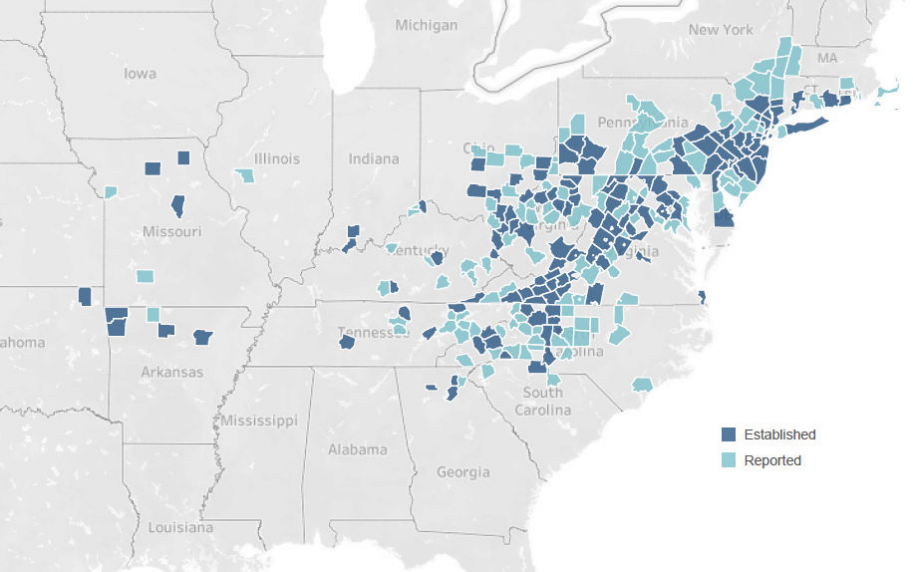
United States Department of Agriculture Animal and Plant Health Inspection Service (USDA APHIS). Counties with established and reported *Haemaphysalis longicornis* in the US as of September 2024. Established populations are those where six or more individual ticks or at least two of the three host-seeking life cycle stages (larva, nymph, adult) have been detected in 1 year. Reported cases are those where a single life cycle stage is present with no consistent collections over time and space. Further information is available at the USDA APHIS website (24).

Of additional concern is the spread of the invasive tick, *Haemaphysalis longicornis*, commonly known as the Asian longhorned tick. As the name implies, it is native to eastern Asia where it is an important human and veterinary pathogen. While *H. longicornis* had been intercepted multiple times at US ports of entry over the years, its detection on a sheep in New Jersey in 2017 marked the first time it was detected outside of a US quarantine station (23). Despite intensive elimination and control efforts, *H. longicornis* has expanded rapidly throughout the eastern and central US, and there are now confirmed populations in 22 states as of September 2024 (Fig. 3) (24). A 2019 study using ecologic niche models predicted that *H. longicornis* could occupy an even broader region of the US, including the southeastern, midwestern, and Pacific northwestern states (25). Female *H. longicornis* ticks can reproduce parthenogenetically (i.e., without a male), resulting in massive animal infestations with thousands of tick progeny and severe associated blood loss. In China and Japan, it serves as a human disease vector for severe fever with thrombocytopenia syndrome virus (SFTSV), a cause of hemorrhagic fever, and *Rickettsia japonica*, the cause of Japanese spotted fever. Additionally, *H. longicornis* may host various *Anaplasma*, *Babesia*, *Borrelia*, *Ehrlichia*, and *Rickettsia* species, raising concerns that it could vector closely related bacteria species in the US. Recently, Price and colleagues detected DNA from the human pathogenic variant of *Anaplasma phagocytophilum* and *Borrelia burgdorferi* in field-collected ticks in Pennsylvania (26). Laboratory studies showed that *H. longicornis* larvae could acquire *B. burgdorferi* during feeding but did not retain infection after molting to the nymphal stage (27). However, there is the hypothetical risk that *H. longicornis* ticks can become infected by taking a partial blood meal, followed by reattaching to a new host and transmitting infection before molting to the next life cycle stage. In support of this hypothesis, Parise et al. demonstrated that *H. longicornis* could acquire *B. burgdorferi* infection during a partial feeding (28). Laboratory studies have also demonstrated that *H. longicornis* can acquire and transmit *Rickettsia rickettsii* under laboratory conditions (29). Lastly, there is concern that *H. longicornis* could serve as a vector for Heartland virus, as it is closely related to SFTSV. Heartland virus has been shown to be experimentally transmissible to mice by *H. longicornis* and transovarially transmitted among *H. longicornis* in a laboratory setting (30). To date in the US, *H. longicornis has* been found on a wide variety of livestock, wildlife, and pets and has been observed biting humans (31). Additional studies are needed to better understand its ability to vector the various tick-borne pathogens found in the US, establish its preferred hosts and feeding habits, and monitor for the presence of imported pathogens vectored by *H. longicornis*, such as SFTSV (32).

The distribution of tick vectors will likely continue to transform with changes in climate, land use, and human behavior (33, 34). Ticks spend most of their time off the host, and their survival, duration of development, and host-seeking behavior are highly influenced by temperature, humidity, and precipitation. Warmer temperatures generally promote tick survival, increase reproductive capacity, lengthen seasonality, and stimulate host-seeking behavior (34). Global warming may, therefore, increase the range of suitable tick habitats in the northern part of the US and southern regions of Canada, as well as lengthen the period that ticks are active (33). However, extreme weather events, high temperatures, and arid conditions may reduce tick survival and biting activity in the southern and southwestern states (34). Tick population expansion is also dependent on the availability of suitable habitat and hosts, including small and large mammals. The ongoing expansion of *I. scapularis* in the Northeast, Upper Midwest, and Ohio Valley regions may be attributed primarily to the expansion of white-tailed deer (*Odocoileus virginianus*) populations following deer conservation efforts and reforestation of former agricultural areas (35, 36). Forest fragmentation leading to a predominance of hosts favored by *I. scapularis*, such as deer and the white-footed mouse, *Peromyscus leucopus*, may also contribute to *I. scapularis* expansion (37).

It is important to note that regional differences in tick densities, host-seeking behavior, and pathogen prevalence significantly influence the risk of human disease. Although *I. scapularis* is broadly distributed across the eastern US, *B. burgdorferi* is detected much more focally within questing ticks (Fig. 2) (38). In the South, infection rates are low largely due to a shift in larval and nymphal host preference from mammals to reptiles, such as skinks and lizards, which are poor reservoirs for *B. burgdorferi* (39). Additionally, southern nymphs rarely quest above the leaf litter likely due to reptilian host preference and hotter, drier conditions, reducing human-tick contact (40). In contrast, northern *I. scapularis* frequently quest above the leaf and feed primarily on small rodents, especially *Peromyscus leucopus*, a highly competent reservoir (40, 41). This results in higher tick infection rates and increased human exposure. Consequently, Lyme disease is far more common in the North than in the South (Fig. 1)—a pattern not predicted by tick distribution alone. Additionally, genomic analysis supports the separation of *I. scapularis* into genetically distinct southern and northern populations (42). It is the latter population that is driving the current Lyme disease epidemic.

## UPDATES IN IMPORTANT TICK-BORNE DISEASES

There have been several important advances in our understanding of tick-borne diseases during the past decade. The following is a brief discussion of common bacterial, viral, and parasitic TBDs and our current understanding of their epidemiology, clinical manifestations, and treatment. It is important to note that many diseases have similar presenting symptoms, such as fever, myalgia, arthralgia, headache, and rash, along with leukopenia, thrombocytopenia, and elevated liver enzyme levels, which may raise a broad differential diagnosis, including other zoonotic infections. Thus, laboratory testing is often important for determining the suspected pathogen. Other notable similarities among TBDs are a lack of licensed vaccines for human use and a lack of specific treatment for viral infections. The CDC’s website on TBDs (https://www.cdc.gov/ticks/about/index.html) and Reference Manual for Healthcare Providers (43) provide additional information.

### Anaplasmosis

Anaplasmosis is due to the obligate intracellular bacterium, *A. phagocytophilum*, previously known as *Ehrlichia phagocytophilum* and *E. equi* (44). Anaplasmosis is the second most common TBD vectored by *I. scapularis*, and concurrent cases of LD have been reported (45). Transfusion- and transplantation-associated disease has also been described (46–48). The number of cases has increased significantly since the disease became nationally notifiable in 1999. In 2021, the highest incidence per million population was in Maine, New Hampshire, Rhode Island, Wisconsin, Massachusetts, Minnesota, and New York (45). Anaplasmosis is a febrile illness associated with malaise, myalgia, severe headache, and gastrointestinal symptoms (43). Rash is uncommon, being present in less than 10% of cases (43). When a rash is noted, it is important to consider potential co-infection with LD. Leukopenia, thrombocytopenia, and elevated liver enzyme levels are common, but a large cohort study showed that the classic triad of leukopenia, thrombocytopenia, and transaminitis was only observed in 24% of cases (49). Doxycycline is the drug of choice for anaplasmosis in patients of all ages, including children (50). Treatment should not be delayed while awaiting laboratory results, as in rare cases, infection may be fatal (50). Fortunately, severe and life-threatening disease is less common compared with other rickettsial diseases, including *Ehrlichia chaffeensis* ehrlichiosis and Rocky Mountain spotted fever (43).

### Ehrlichiosis

Ehrlichiosis is primarily due to infection with the intracellular bacteria, *Ehrlichia chaffeensis* and *E. ewingii* (43). These two bacteria are transmitted by *A. americanum*, and infection is found predominantly in the Southeast and South Central US, from the east coast extending westward to Texas (51). Rare cases of transfusion and transplantation-associated cases have also been described (48). Human ehrlichiosis had been recognized in the 1980s but only became a reportable disease in 1999 (43). From 2017 through 2021, the states with the highest incidence of reported *E. chaffeensis* ehrlichiosis cases were Missouri, Arkansas, North Carolina, New York, and Tennessee (43). In 2009, the third cause of human ehrlichiosis, *Ehrlichia muris eauclairensis*, was detected from whole blood of individuals with exposure to tick bites in the Upper Midwest (7). Unlike the other causes of human ehrlichiosis in the US, *E. muris eauclairensis* is transmitted by *I. scapularis*, thus adding to the growing list of organisms transmitted by this tick (52, 53). There have been more than 200 cases of *E. muris eauclairensis* detection since the initial report (unpublished Mayo Clinic data). In 2008, the ehrlichiosis case definition for reportable diseases was split into *E. chaffeensis* infection, *E. ewingii* infection, and undetermined ehrlichiosis/anaplasmosis, the latter of which includes *E. muris*. Unfortunately, there is significant serologic cross-reactivity among the three agents, so species-specific reporting relies on the use of molecular-based tests, which have limited availability. The number of total ehrlichiosis reported cases has increased steadily, from 200 cases in 2000 to 2,093 cases in 2019 (43).

Clinical symptoms are similar to those of anaplasmosis, with fever, chills, headache, malaise, myalgia, and gastrointestinal symptoms (43). Rash is more common than seen with anaplasmosis and may be present in 60% of children and 30% of adults (54). Leukopenia, thrombocytopenia, and elevated liver enzyme levels are common. *E. chaffeensis* can cause fatal disease, whereas no fatalities have been reported to date for *E. ewingii* and *E. muris eauclairensis* infection (43). Immunocompromised patients, as well as young children and individuals > 70 years may be at increased risk of severe disease. Treatment is with doxycycline for patients of all ages, including children, and should not be held while awaiting laboratory test results (43).

### Lyme disease

Lyme disease, also known as Lyme borreliosis, is the most common tick-borne disease in the US. LD is caused by members of the *Borrelia burgdorferi sensu latu* (Bbsl) complex. *B. burgdorferi sensu strictu* (hereafter referred to as *B. burgdorferi*) is the primary cause of LD in the US and is transmitted to humans through the bite of an infected *I. scapularis* or *I. pacificus* tick (1). A significantly smaller number of cases is also caused by *B. mayonii* in the Upper Midwest (9). Other members of the Bbsl complex occasionally detected in humans are *B. bissettii*, *B. americana*, and *B. andersoni*, but further study is needed to understand the significance of these findings (18). Of note, there was a 2014 proposal to move the Bbsl into a new genus, *Borreliella*, based on genetic and biological differences between the Bbsl and other *Borrelia* clades, such as the relapsing fever borreliae (55). However, this taxonomic scheme has not been widely accepted by bacteriologists (56), and this article will retain the *Borrelia* genus designation for members of the Bbsl complex.

National LD surveillance began in the US in 1991 and has shown a continual increase in reported cases to present day. A revised case definition went into effect January 1, 2022, allowing for case reporting based on laboratory evidence alone in high incidence jurisdictions without the requirement for associated clinical information (3). This alteration resulted in a sharp increase in the number of reported cases that year, with 62,551 reported cases—1.7 times the annual US average of 37,118 during 2017–2019 (3). As noted previously, the actual incidence is thought to be eight to 12 times higher than reported (3).

Typical symptoms of LD include fever, fatigue, headache, myalgia, arthralgia, lymphadenopathy, and a rash, called erythema migrans (43, 57). Erythema migrans (EM) occurs at the site of the tick bite and may be annular or homogeneous, expanding over several days. The classically described appearance is a targetoid rash with a central clearing, but this is not always present. EM is a diagnostic feature of LD in endemic regions but will not be seen in ~30% of patients, and its absence cannot be used to exclude the diagnosis (43). The most common laboratory abnormality is elevated erythrocyte sedimentation rate. Unlike many other TBDs, leukopenia, thrombocytopenia, and elevated liver enzyme levels are less common and, when present, may indicate co-infection with other *I. scapularis-*transmitted pathogens (58–60).

If untreated, early localized infection will disseminate in approximately 60% of patients to cause multiple EM rashes, neurologic, ocular, rheumatologic, and cardiac manifestations. Conduction abnormalities associated with LD carditis may rarely be fatal (43). Most patients fully recover following antibiotic therapy, but up to 10% may experience prolonged fatigue, myalgias, and cognitive impairment. The preferred term for this condition is post-treatment Lyme disease syndrome as the etiology is currently unknown. There is no clear evidence of ongoing infection, and the provision of longer-term antibiotic therapy has not been shown to provide additional benefit over the standard course (61). A similar clinical phenomenon has now been recognized following coronavirus disease (COVID-19) (i.e., “long COVID”).

### *Borrelia miyamotoi* borreliosis

Unlike members of the Bbsl complex, *B. miyamotoi* belongs to the relapsing fever clade of the *Borrelia* genus and is, therefore, more closely related to the soft-tick transmitted RF borreliae. *B. miyamotoi* is found throughout the Northern Hemisphere, including North America, Europe, and Asia (62). In the US, *B. miyamotoi* is found in the Northeast and Upper Midwest regions, where it is vectored by *I. scapularis*, and on the West coast, where it is vectored by *I. pacificus* (63). Interestingly, tick prevalence studies show that the prevalence of *B. miyamotoi* carriage exceeds that of *B. burgdorferi* in *I. pacificus* (63). In contrast to *B. burgdorferi*, *B. miyamotoi* is capable of transovarial transmission—meaning it can infect the developing eggs within a gravid female tick—as well as transstadial transmission, where the pathogen is carried from one life stage to the next (such as from larva to nymph or nymph to adult). This allows *B. miyamotoi* to be at least partially sustained within tick populations through vertical transmission, and larvae may serve as potential vectors for the bacterium (64). *B. miyamotoi* borreliosis is not currently a nationally notifiable disease in the US; thus, the incidence of infection is unknown. However, *B. miyamotoi* borreliosis appears to be significantly less common than LD and anaplasmosis, with only 101 cases published between 1991 and 2019 in the US (65). A large 2020 analysis of 13,038 residual clinical specimens (87.4% EDTA blood) from patients with suspected tick-borne infections in the US detected 24 instances of *B. miyamotoi* out of 881 specimens testing positive for bacterial tick-borne agents (53). In comparison, 498 specimens were positive for *A. phagocytophilum*. In another insightful study, 8,575 PCR tests performed for both *B. burgdorferi* and *B. miyamotoi* in a highly Lyme disease-endemic region of New York (Stony Brook Medicine System, Suffolk County) revealed positive results in 19 (0.2%) and 17 (0.19%), respectively (66). *Anaplasma phagocytophilum* PCR performed during the same time period had 0.4% positivity (80/17501).

The clinical presentation of *B. miyamotoi* borreliosis varies with the immune status of the host. In immunocompetent hosts, infection commonly presents with non-specific, short-lived flu-like symptoms, including fever, chills, fatigue, myalgias, and arthralgias (62, 67). Leukopenia, thrombocytopenia, and elevated liver enzyme levels are common (68). Despite being a member of the relapsing fever clade, relapsing febrile episodes are rare (62). Immunocompromised patients may experience severe disease associated with meningoencephalitis with reduced cognition, confusion, disturbed gait, uveitis, iritis, and hearing loss (67). Doxycycline is the preferred antibiotic treatment for adults without neurological complications, whereas ceftriaxone is preferred for patients with meningoencephalitis (65). Symptoms usually resolve within a week of starting antibiotic therapy (62).

### Rocky Mountain spotted fever

Rocky Mountain spotted fever (RMSF) is caused by the gram-negative, obligate intracellular bacterium, *Rickettsia rickettsii*. It is the most common rickettsial infection in the US and is most prevalent in the southeastern and south-central states, as well as the southwestern states bordering northern Mexico. Greater than 60% of cases are reported in Arkansas, Missouri, North Carolina, Oklahoma, and Tennessee, where the organism is transmitted primarily by *Dermacentor variabilis* (a.k.a. American dog tick and wood tick) and *D. andersoni* (Rocky Mountain wood tick). In contrast, transmission of *R. rickettsii* in Arizona and Northern Mexico is primarily by the brown dog tick, *Rhipicephalus sanguineus*, which can be found in high numbers on domestic dogs and in the peridomestic habitat (69). RMSF is a nationally notifiable disease in all states, except Alaska and Hawaii (50, 70). Less-pathogenic rickettsial species, including *R. parkeri* and *R. philipii* (formerly 364D), can also infect humans, and infection with these entities and RMSF cannot be differentiated using serologic tests. Therefore, the Council for State and Territorial Epidemiologists changed the name of the notifiable condition to spotted fever group (SFG) rickettsiosis in 2009 to encompass this broad group (70). The reported number of cases rose from 495 cases in 2000 to 6,248 cases in 2017 (69). However, the case definition was updated in 2020 to more accurately capture true, acute cases of SFG rickettsiosis, resulting in a significant drop in reported cases.

RMSF is a serious disease that can cause end-organ damage and death if not rapidly treated. Initial symptoms include fever, chills, headache, malaise, and myalgia (50). Nausea, vomiting, photophobia, abdominal pain, and anorexia may also be present. The characteristic petechial rash due to systemic vascular injury, which is present in 90% of cases, typically appears 2–4 days after the onset of fever and may, therefore, not be present at the time the patient seeks care (50). Thrombocytopenia, elevated liver enzymes, and normal to slightly elevated white blood cell count with bandemia are common (50). Early empiric therapy is indicated given the potential for severe outcomes. Doxycycline is the treatment of choice for adults and children (50).

### Soft tick relapsing fever borreliosis

Soft tick relapsing fever (STRF) is a rare but potentially serious disease caused by *Borrelia* spirochetes in the relapsing fever clade and transmitted by soft-bodied ticks in the genus *Ornithodoros* (16). *Ornithodoros* ticks can live for decades and, once infected, can transmit the infection to humans throughout their lifetime. The most common causes of STRF in the US are *B. hermsii* and *B. turicatae* (71). Unlike ixodid (hard-bodied) ticks, soft ticks are rarely seen, as they feed rapidly and then quickly detach and scatter. People usually become infected in mountainous regions of western states when staying overnight in seasonal, rodent-infested cabins where *Ornithodoros hermsii* are sheltering. Ticks emerge at night and briefly feed on sleeping inhabitants, transmitting *B. hermsii* in the process. Less commonly, people become infected with *B. turicatae* from *Ornithodoros turicata* while exploring caves in Austin, Texas (71, 72). During 2012–2021, 251 STFR cases were identified in 11 of the 12 states in which infection is reportable (16).

Patients with STRF most commonly present with high fever, chills, headache, and myalgias (16). Fever subsides as the host immune response clears most of the spirochetes from the bloodstream. However, if untreated, febrile episodes may recur every 7–10 days due to the spirochetes’ ability to change its outer surface protein antigens and evade the host’s immune response (73). This allows the *Borrelia* population to once again expand in the bloodstream, resulting in fever. Complications of infection include neurologic disease, myocarditis, acute respiratory distress syndrome, and pregnancy loss in infected women (16). Of the 211 STFR cases identified during 2012–2021 for whom hospitalization data were available, 115 (55%) were hospitalized with no deaths documented (16). Treatment with doxycycline, azithromycin, or beta-lactam antibiotics should be provided promptly while awaiting laboratory results to avoid complications.

### Tularemia

Tularemia is a rare, potentially fatal, nationally notifiable disease caused by the gram-negative bacterium, *Francisella tularensis* (74, 75). The two subspecies that cause human disease in the US are subsp. *tularensis* (type A) and subsp. *holarctica* (type B) (76). This highly infectious, tier-1 select agent has been reported in all states, except Hawaii, and can be transmitted to humans through bites of a wide variety of infected arthropods, including hard ticks, deer and horse flies, and mosquitoes (77). Ticks most commonly implicated in *F. tularensis* transmission are *D. andersoni*, *D. variabilis*, and *A. americanum*. Other means of transmission include inhalation of contaminated aerosols, drinking contaminated water, and improper handling of infected animals (74, 75) (78). Through 2022, 2,462 probable and confirmed cases were reported from 47 states, with just four states (Arkansas, Kansas, Missouri, and Oklahoma) accounting for 50% of cases (74). This represents a 56% higher incidence of tularemia than reported during 2001–2010, reflecting an increased number of reported probable cases, and possibly improved case detection methods and/or increased human cases. The highest incidence of cases was in children ages 5–9, adult male ages 65–84, and American Indian/Alaska Native individuals (74).

Clinical presentation varies by the route of infection. Of the 1,163 reported US cases during 2006–2021, 42 and 16.1% were ulceroglandular and glandular, respectively (79). Ulceroglandular disease is characterized by a skin ulcer at the site of bacterial entry (e.g., tick bite) with associated regional lymphadenopathy, whereas glandular disease is similar but lacks the ulcer. Other classic forms of disease include oropharyngeal, pneumonic, typhoidal, and oculoglandular. Rare manifestations include endocarditis, encephalitis, meningitis, osteomyelitis, peritonitis, and septic arthritis (80, 81).

The average fatality rate is <2% in the US but is significantly higher for certain type A genotypes. Antibiotic therapy is essential for minimizing complications and fatalities. A recent analysis of antimicrobial treatment patterns and illness outcomes from reported cases of tularemia during 2006–2021 found that aminoglycosides, fluoroquinolones, and tetracyclines were independently associated with increased odds of survival (79).

### Powassan virus disease

Powassan virus (POWV) is a single-stranded, positive-sense RNA virus in the family Flaviviridae, genus *Orthoflavivirus* with two lineages. Lineage I is transmitted by *Ixodes cookei* and *I. marxi*, while lineage II (also known as deer tick virus) is transmitted by *I. scapularis* (43). POWV disease has the lowest number of annual cases and disease incidence of the *I. scapularis* transmitted diseases that are nationally notifiable, with only 42 cases reported as of 17 September 2024 (82). Of these, 42 were cases of neuroinvasive disease, suggesting that only the most severe cases come to medical attention. The true number of cases is likely underappreciated given the lack of widely available diagnostic tests and limited physician knowledge regarding the pathogen. Most cases have been reported from the Northeast and Upper Midwest, similar to the distribution of other *I. scapularis-*transmitted pathogens (43). Symptoms include fever, headache, nausea, vomiting, and general weakness. Leukopenia, thrombocytopenia, and elevated liver enzyme levels may be seen. Patients with neuroinvasive disease have signs and symptoms of meningoencephalitis with meningeal signs, seizures, altered mental status, paresis, cranial nerve palsies, or movement disorders (43). Approximately one out of 10 people with neuroinvasive disease die, and approximately half of survivors have ongoing neurologic manifestations. Treatment is supportive only (43).

### Babesiosis

Babesiosis is the third most common *I. scapularis*-transmitted disease in the US and is due primarily to *B. microti*, an intraerythrocytic apicomplexan parasite (83). A much smaller number of cases due to *B. duncani* in the western Pacific states have also been reported, as well as rare cases of infection with a *B. divergens*-like pathogen in Missouri and, recently, Michigan (84). A recent CDC review of reported babesiosis cases from 2011 to 2019 showed a significant increase in babesiosis incidence in several northeastern states during this time period, with the highest incidences reported from Rhode Island, Maine, and Massachusetts (85). Notably, Maine, New Hampshire, and Vermont were not previously considered to be endemic for babesiosis but had similar or even greater reported incidences in 2019 than known endemic states in the Northeast. Based on these data, the CDC now considers these three states to be endemic for babesiosis (85). The states with the highest number of cases overall were New York, Massachusetts, and Connecticut. *Babesia* species may also be transmitted by blood transfusion, organ transplantation, and perinatally. As transfusion-transmittable diseases, the expansion of babesiosis may have implications for the nation’s blood supply. The Food and Drug Administration (FDA) recommends testing each donation collected in 14 states (Connecticut, Delaware, Maine, Maryland, Massachusetts, Minnesota, New Hampshire, New Jersey, New York, Pennsylvania, Rhode Island, Vermont, Virginia, and Wisconsin) and the District of Columbia for *Babesia* using the recently FDA-approved antibody and nucleic acid amplification tests (86, 87)

Babesiosis presents with a broad spectrum of manifestations, from asymptomatic infection to severe, life-threatening disease. An estimated 50% of infected children and 25% of adults are asymptomatic. When present, clinical manifestations occur 1–4 weeks after a tick bite or 1–9 weeks after a contaminated blood transfusion and include fever, malaise, myalgia, arthralgia, headache, and gastrointestinal symptoms (43). Hepatosplenomegaly, as well as jaundice and dark urine due to erythrocyte lysis, may also be noted. Typical laboratory test abnormalities include hemolytic anemia, thrombocytopenia, and elevation of liver enzymes. Manifestations of severe diseases include disseminated intravascular coagulation, renal failure, acute respiratory distress, hemodynamic instability, altered mental status, and death (43). Risk factors for severe diseases include immune compromise and asplenia (83). Like anaplasmosis, babesiosis may co-exist with LD and potentially result in increased disease severity (88). Treatment decisions should consider multiple factors, such as patient age, immune status, splenic function, and pregnancy status. In immunocompetent adults, the preferred drug regimen is azithromycin and atovaquone, with an alternate regimen of clindamycin and quinine (39G). Immunocompromised and asplenic patients may require higher and prolonged doses.

## GENERAL APPROACHES TO LABORATORY DETECTION OF TICK-BORNE DISEASES

The field of clinical microbiology continues to evolve, with novel diagnostic assays and methods developed and implemented into clinical practice at a rapid pace. In light of these advances, it is important for laboratories to routinely review longstanding diagnostic approaches and determine whether changes or updates to currently offered testing are warranted. With respect to VBDs, a combination of traditional diagnostic tests (e.g., microscopy, serology, nucleic acid amplification testing [NAAT]) remains the preferred approach for many of the target pathogens, while the use of more contemporary methods (e.g., targeted and shotgun metagenomic next-generation sequencing [mNGS]) is increasingly considered (Table 2). A multi-pronged diagnostic strategy, with the consideration of possible co-infections, remains necessary for many of the VBDs primarily due to the non-specific and overlapping symptoms associated with these pathogens, which also have large overlapping regions of endemicity.

**TABLE 2:** Preferred diagnostic testing approaches for vector-borne diseases in North America^*e*^

Disease	Preferred diagnostic method(s)^*a*^	
	≤7 days of symptoms	>7 days of symptoms
Anaplasmosis	NAAT (preferred) Peripheral blood smear Baseline serology	Serology (IgG preferred) NAAT (decreased sensitivity with time)
Arboviruses	Baseline serology NS1 antigen (dengue virus) NAAT	Convalescent serology (as needed)^*d*^
Babesiosis	NAAT (preferred) Peripheral blood smear^*b*^ Baseline serology	Peripheral blood smear^*b*^ NAAT Serology (IgG preferred)
Ehrlichiosis	NAAT (preferred) Peripheral blood smear Baseline serology	Serology (IgG preferred) NAAT (decreased sensitivity with time)
Lyme disease	Baseline serology (preferred) NAAT^*c*^	Convalescent serology (as needed) NAAT^*b*^
Rickettsioses	Baseline serology (preferred) NAAT	Convalescent serology (as needed)
Tick-borne relapsing fever	NAAT (preferred) Peripheral blood smear	NAAT (preferred) Serology
Tularemia	Culture NAAT	Culture Serology (IgM and IgG)

^*a*^ The preferred method may vary with local test availability. Metagenomic next-generation sequencing may be useful for diagnosis of tick-borne and other zoonotic pathogens when routine testing is unrevealing.

^*b*^ Peripheral blood smear is preferred in areas without access to NAAT and to determine percent parasitemia.

^*c*^ Highest sensitivity of NAAT for *B. burgdorferi* is in synovial fluid and erythema migrans tissue. Limited utility of NAAT for diagnosis of acute Lyme disease caused by to *Borrelia burgdorferi*, but NAAT is the test of choice for detecting Lyme disease caused by *Borrelia mayonii* (endemic to the Upper Midwestern US).

^*d*^ Serologic testing for arboviral pathogens is typically performed using ELISA methods, which have limited specificity. Positive results are frequently confirmed by PRNT at select public health laboratories.

^*e*^ Abbreviations: EM—erythema migrans; NAAT—nucleic acid amplification test.

**Fig 4:**
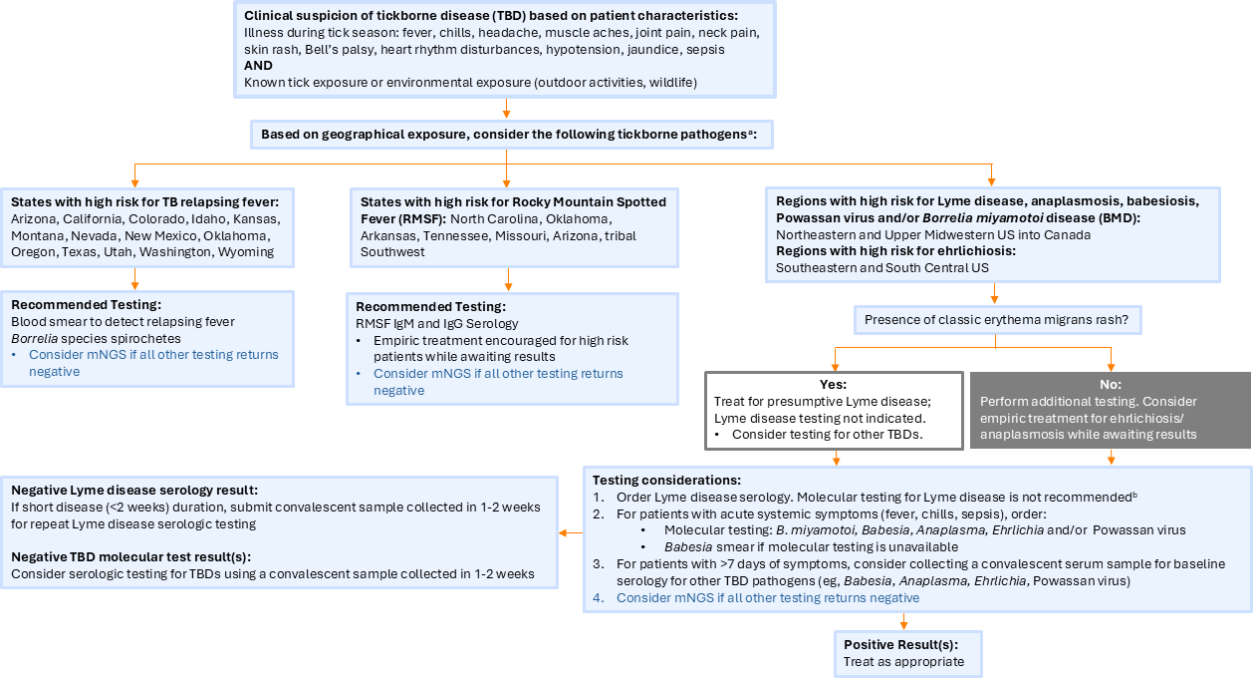
Diagnostic algorithm for tick-borne disease diagnostic testing in North America. This suggested algorithm includes the most common TBDs for which academic and/or reference laboratories in North America offer testing (**a**). For less common pathogens, including Bourbon virus, Heartland virus, and Colorado tick fever, contact your local public health laboratory or the CDC to discuss testing options. Molecular testing for *Borrelia* species causing Lyme disease using blood, plasma, or serum is not recommended (**b**). Molecular testing for Lyme disease is most useful for testing synovial fluid and tissue biopsies of suspected erythema migrans rashes. Abbreviations: mNGS, metagenomic next-generation sequencing

With the availability of numerous diagnostic methods for the same vector-borne pathogen comes the challenge of maintaining optimal test stewardship to ensure that the preferred test is used for the right patient at the appropriate time in their disease course. Creating a diagnostic testing algorithm, which considers all of the aforementioned factors and is available to reference at the time of test order placement, is one approach to maintain diagnostic test stewardship, which can benefit both patient-facing healthcare workers and the clinical microbiology laboratory. As an example, Fig. 4 is the TBD diagnostic testing algorithm currently used at Mayo Clinic to help guide appropriate test ordering. Briefly, healthcare workers are first reminded to consider TBDs based on patient symptoms, general laboratory findings, time of year, and outdoor geographic exposure history, and, based on the latter, are directed toward the consideration of specific TBD pathogens (e.g., consider RMSF for patients with exposure in North Carolina, Oklahoma, Arkansas, Tennessee, Missouri, Arizona, and the Tribal Southwest). Based on this initial direction, the algorithm then provides recommended initial diagnostic test(s) to order, as well as follow-up tests that should be considered based on both the duration of patient symptoms, which can impact which test is recommended (i.e., molecular or serology), and the risk of co-infections. Below, we provide a review of the classic, contemporary, and novel diagnostic approaches, which are currently utilized for the detection of TBDs.

### Microscopy

The microscopic examination of peripheral blood smears can be used to diagnose a number of TBDs, although performance characteristics of this method vary widely by pathogen, and knowledge of the limitations should dictate the most appropriate diagnostic workflow. Microscopy of Giemsa-stained thin and thick blood films is the gold standard for diagnosis of *Babesia* species (83). Pathogen identification, initial percent parasitemia, and monitoring of the parasitemia in response to therapy can all be performed with microscopy. Morulae, microcolonies of bacteria in the cytoplasm of infected cells, can be seen on blood smears for patients infected with *A. phagocytophilum* or *Ehrlichia* species. Recognition of *A. phagocytophilum* and *E. ewingii-*infected granulocytes or *E. chaffeensis-*infected monocytes is diagnostic. However, microscopic detection is insensitive and is, therefore, not a recommended stand-alone diagnostic method: 25–75% for *A. phagocytophilum* and ~3% *Ehrlichia* (89).

Finally, some *Borrelia* species can also be detected via blood smear. These primarily include the causative agents of tick-borne relapsing fever, *B. hermsii*, *B. turicatae*, and *B. parkeri*, which reach sufficient bacterial burdens in peripheral blood to be detected microscopically (90). *B. mayonii*, a less frequent cause of Lyme disease, has rarely been reported to be seen on blood films (91). This contrasts with *B. burgdorferi*, the primary cause of Lyme disease in the US, which has not been detected with blood film microscopy. *B. miyamotoi* is also unlikely to be detected in peripheral blood films (92), although spirochetes have been occasionally detected in blood and CSF in cases of high density (93–95). In recent years, artificial intelligence (AI) has been successfully applied to digital images to detect a variety of microorganisms, including *Babesia* in blood films (96). Applications of AI-assisted microscopy are rapidly expanding and will likely play an important role in the diagnosis of applicable TBDs.

### Nucleic acid amplification

Nucleic acid amplification tests (NAATs), including those based on polymerase chain reaction (PCR), are useful acute-phase diagnostics for some TBDs. However, to date, no FDA-approved or -cleared diagnostic molecular assays exist for the detection of TBD pathogens for clinical diagnosis. In response, some laboratories have developed, validated, and implemented laboratory-developed tests (LDTs), an important diagnostic development pathway with associated uncertainties related to a recently introduced FDA rule (97). However, the FDA rule was vacated by the US District Court for the Eastern District of Texas, determining that it exceeds the authority granted to the agency in the Food, Drug, and Cosmetic Act (https://www.acla.com/federal-court-vacates-fda-rule-on-laboratory-developed-testing-services-siding-with-acla/). The Department of Health and Human Services, Department of Justice, and FDA could pursue additional next steps that may impact FDA oversight, including an appeal of the ruling. Additionally, Congress could choose to create new legislation for LDT regulation that may be administered by FDA or Clinical Laboratory Improvement Amendments. Therefore, laboratories employing NAATs for diagnosis of TBDs should monitor the situation so that they remain in compliance with all applicable requirements.

The most common applications of NAATs for detecting tick-borne bacteria include *Anaplasma* and *Ehrlichia* from whole blood, which is the recommended diagnostic approach within the first week of symptom onset (98, 99). NAATs are also available for the detection of soft- and hard-tick relapsing fever *Borrelia* species from whole blood during acute infection (8, 100). For the LD-causing *Borrelia*, whole blood NAAT may be useful in acute-phase diagnosis of *B. mayonii*, given the increased spirochetemia when compared to *B. burgdorferi* (101). However, given the limited number of cases of *B. mayonii*, the true nature of this sensitivity advantage is unknown. Whole blood NAAT, as well as molecular detection from CSF, is poorly sensitive for *B. burgdorferi* due to low levels of circulating DNA (below the limit of NAAT detection) and has little to no clinical utility. The best performance for *B. burgdorferi* NAAT has been established from synovial fluid (102).

Use of molecular testing for tick-borne viruses is limited by the short viremic window during infection, which has commonly elapsed by the time patients present for care. An extended window of utility may exist in immunocompromised patients who may have delayed clearance of the virus and in whom antibody production may be stunted. Recent advancements include the addition of urine as an alternative sample source for POWV NAAT, with studies showing that testing of urine extends the window of utility for detection of POWV. Prolonged viral shedding in urine has been established for other flaviviruses as well, including the West Nile and Zika viruses (103).

A small number of laboratories offer diagnostic *Babesia* PCR. Applications are limited given the longer turnaround time compared to microscopy but can be useful in cases of diagnostic uncertainty. CDC recently updated guidance in response to cases of endemic malaria to recommended confirmatory *Plasmodium* PCR and *Babesia* PCR in cases where *P. falciparum* and *Babesia* species cannot be reliably differentiated (104). The most widespread use of *Babesia* PCR, and the only example of a TBD FDA-approved molecular test, is for blood donor screening. The FDA approved the first *Babesia* NAAT for screening of the blood supply in 2018 (87), with two additional approvals since (105). In 2019, the FDA recommended screening of blood donors using *Babesia* NAATs in 14 high-risk states (86). This approach has been shown to be effective in reducing the number of transfusion-transmitted babesiosis cases (106).

If an attached tick is removed, tick identification is recommended, as details including the species and life cycle stage can inform conversation about potential pathogen exposures, symptom monitoring, or the need for prophylaxis. The Infectious Diseases Society of America, American Academy of Neurology, and American College of Rheumatology recommend against testing ticks using NAATs to determine if they harbor a pathogen (107). Carriage of a potential pathogen does not equate to transmission, nor does it predict infection, and, as a result, can lead to unnecessary treatment.

### Next-generation sequencing

Recently, next-generation sequencing applications, both targeted and metagenomic, have found use in the detection of TBD. Both methods find advantages in their ability to cast a broad net in testing, detecting both TBDs and non-TBDs with similar symptoms or presentations. Two published targeted NGS (tNGS) assays targeting the 16s rRNA gene of bacteria showed the ability to detect a broad range of tick-borne bacteria in addition to similarly presenting zoonotic infections (e.g., *Coxiella burnetii* and *Leptospira* species) and the promise in detecting underappreciated or emerging pathogens (53, 108). The CDC and New York State Public Health Laboratory have also reported a “Universal Parasite Diagnostics Assay” (UPDx) based on the NGS metabarcoding of 18S rRNA that has been successfully used to detect known and novel *Babesia* strains (109, 110). Lastly, unbiased (“shotgun”) metagenomic NGS (mNGS) from plasma microbial cell-free DNA has shown the ability to detect a wide array of zoonotic pathogens, including tick-borne bacteria (111). The same assay was evaluated for the detection of *B. burgdorferi* in two separate studies (112, 113). The performance was encouraging, albeit using a research-only threshold for reporting below the clinically available reportable threshold, for a pathogen that is often difficult to detect from blood via molecular methods. Further study and refinement are needed to determine the full utility of plasma cfDNA mNGS for the detection of tick-borne bacteria (114). A recently released pre-print has described the utility of mNGS from CSF for the detection of vector-borne viruses (115). While most commonly diagnosed by serology, these methods suffer from specificity challenges and variable performance in immunocompromised patients. Detection with mNGS provides an alternative method for diagnosis and may contribute to a better understanding of these hard-to-differentiate or less commonly investigated viruses.

### Serology

Serologic testing has been a longstanding approach for the diagnosis of TBDs; however, with the development and increasing availability of NAATs, serology is no longer the preferred method for detection of acute disease caused by a number of these pathogens. Multiple limitations associated with serologic testing have been described, regardless of targeted antibody class, including prolonged time to seroconversion, which often occurs beyond the acute phase of disease. Studies assessing the kinetics of IgM/IgG antibody development against *A. phagocytophilum*, *Ehrlichia* species, and *Babesia* species generally show seroconversion occurring 7 to 14 days after symptom onset, limiting the clinical utility of this approach in patients presenting with acute disease (116–119). As a result of this inherent biologic delay, reliance on a positive IgM result to diagnose acute infection due to either of these tick-borne agents is generally not recommended; instead, a NAAT performed on whole blood should be ordered for patients presenting with acute disease. While some patients may exhibit high pathogen-specific IgG titers (≥1:640) early in disease onset, which would be consistent with a presumptive diagnosis, the majority of acutely ill patients who are only tested for IgG-class antibodies result as IgG-seronegative or with a low-titer positive result (i.e., ≤1:128) (43, 119). In the latter two scenarios, repeat testing on a convalescent sample collected in 2–3 weeks is necessary to either definitively rule out (i.e.*,* IgG remains negative) or establish (i.e.*,* document a fourfold or higher change in titers) a diagnosis. Due to the need for convalescent sample collection, however, this is considered a retrospective diagnostic approach and not ideal for acute patient care. Result interpretation for IgG-class antibodies is further complicated by the issue of antibody persistence, which can range from months to years following disease resolution (120, 121). As a result, convalescent testing of initially seropositive patients is important to determine whether detectable IgG is due to prior (<4-fold change in titers) or recent (≥4-fold change in titers) disease. Finally, serologic assays for detection of antibodies to *A. phagocytophilum*, *Ehrlichia*, and *Babesia* species suffer from challenges associated with specificity, including both the potential for false-positive results due to cross-reactivity between closely related species (*A. phagocytophilum*, *E. chaffeensis*, *Coxiella burnetii*, rickettsial species, etc.) and due to the risk of false-negative results as a result of limited cross-reactivity within a genus. The latter is particularly problematic for *Babesia* species, as commercially available assays are typically designed using *B. microti* whole organism or antigens, which do not share significant homology with other endemic *Babesia* species, including *B. duncani* and *B. divergens* (122, 123); this limited cross-reactivity may potentially lead to missed diagnoses if serology is the sole diagnostic method employed.

While many of the limitations associated with serologic testing for anaplasmosis, ehrlichiosis, and babesiosis are widely applicable across pathogens, serology remains either the preferred diagnostic approach or is a key diagnostic component for many other TBDs primarily due to either the limited sensitivity and/or the absence of molecular assays for some of these common pathogens. Among these, the diagnosis of POWV remains reliant on both molecular testing, which is most sensitive in the first 5 to 7 days post symptom onset and, subsequently, on serologic assays for detection of virus-specific IgM antibodies, which are most useful for patients presenting after the first week of symptoms when viremia is typically undetectable. This dual-method approach, which is based on timing post-symptom onset, for POWV is consistent with recommended diagnostic approaches for other common mosquito-borne arboviral pathogens, including the West Nile and dengue viruses and others (124).

Unique among the TBDs, however, are RMSF and LD, for which diagnosis of systemic disease, and in the case of LD, neuroborreliosis, is heavily reliant on serologic evaluation. Although molecular tests for *R. rickettsii* are available through public health laboratories, including the CDC, this pathogen infects endothelial cells lining blood vessels, resulting in few organisms circulating in the bloodstream and, ultimately, low NAAT sensitivity in blood from patients with early or mild disease (125). Antibodies, including both IgM and IgG, against *R. rickettsii* become detectable approximately 7 to 10 days after symptom onset, and, similar to the aforementioned TBDs, definitive diagnosis can be established following documentation of seroconversion and/or a fourfold rise in titers. Importantly, however, due to the high mortality rate associated with RMSF, patients should be started on empiric therapy (e.g., doxycycline) while awaiting serologic test results. Additionally, it is important to note that there is significant cross-reactivity among serologic testing for RMSF and other closely related members of the spotted fever group (78, 126).

Diagnostic testing for LD in North America was first standardized in 1994 during the Second National Conference on Serologic Diagnosis of Lyme Disease, which resulted in the recommendation to use a two-tiered testing approach for all patients with suspected LD (127). This standard two-tiered testing algorithm (STTA) consisted of an initial, sensitive enzyme immunoassay (EIA) or an immunofluorescence immunoassay (IFA), followed by western blot testing for IgM- and IgG-specific antibodies of initially reactive samples. Although some improvements to *B. burgdorferi* serologic assays were made over the years, including the development of both EIAs and immunoblots based on recombinant *B. burgdorferi* proteins and use of densitometry readers to provide objective interpretation of blot banding patterns, the algorithm remained unchanged until July 2019 when the CDC endorsed a modified two-tiered testing algorithm (MTTA) and the FDA-cleared multiple serologic assays for use in a MTTA (128). The key difference between the STTA and MTTA is the replacement of supplemental IgM/IgG immunoblot testing with either total antibody or IgM- and IgG-specific EIAs, which must be based on *B. burgdorferi* antigens different than the first-tier EIA(s) (101). The MTTA has multiple advantages over the STTA, including higher sensitivity among patients with early LD (e.g., erythema migrans), which ranges between 42 and 58% for the STTA and 53 and 67% for the MTTA across studies (129–132). Notable sensitivity of the MTTA at other disease stages is equivalent to the STTA, as is specificity (97–99.5%) across LD mimics and other infections. Other advantages include the ability to detect antibodies against multiple LD-causing *Borrelia* species, which is limited for the STTA due to use of antigens solely from the *B. burgdorferi* B31 strain and restrictive interpretation criteria. The ability to reflex first-tier positive results to secondary EIAs allows smaller laboratories to perform all parts of the algorithm rather than referring samples to reference laboratories, which leads to faster turnaround time and potentially lower cost. Finally, the elimination of immunoblots alleviates the challenges around subjective blot interpretation. The MTTA is not without limitations; however, clinicians are no longer able to monitor or follow the expansion of the IgG antibody response given that EIAs are qualitative in nature. This limits the ability to diagnose potential re-infections or gauge infection stage. Additionally, similar to the STTA, the MTTA cannot (and should not) be used to monitor response to therapy.

#### Lyme neuroborreliosis

Multiple members of the Bbsl complex have been associated with causing neuroinvasive disease, which typically manifests in patients as a triad of symptoms, including meningitis, cranial neuropathy, and painful radiculoneuritis. Although the reported incidence rates of Lyme neuroborreliosis (LNB) vary across geographic regions, the latest studies suggest that up to 12.5% of patients with Lyme disease in the US may develop neurologic disease (133). Diagnosis of LNB requires testing peripheral blood for antibodies to Bbsl, as over 97% of patients with LNB are seropositive, and often necessitates evaluation of CSF for both routine studies (i.e., protein, glucose, and cell count) and intrathecal, Bbsl-specific antibody production. Determination of whether Bbsl-specific antibodies in CSF are produced intrathecally (i.e*.*, in the CNS due to local infection) or whether they are present as a result of passive diffusion across the blood-brain barrier is done through the establishment of an antibody index ratio. Simply put, this ratio compares the level of Bbsl-specific antibodies in CSF versus serum and is normalized to total IgG in both sample sources, which are ideally collected concurrently to minimize the impact of CSF turnover (134). The LNB antibody index is associated with a sensitivity of 70–90% among patients presenting in the first 4–6 weeks post-infection and over 95% at later timepoints (135, 136). Importantly, determining a Bbsl antibody index is currently included in the diagnostic criteria for LNB in the European Federation of Neurological Societies guidelines and recommended by the Infectious Diseases Society of America/American Academy of Neurology/American College of Rheumatology guidelines for patients who have a CSF specimen collected (107). Unfortunately, due to the lack of FDA-cleared assays and the complexity associated with developing and determining a LNB antibody index, only a few laboratories in the US currently offer Bbsl antibody index testing.

## CONCLUSION

TBDs pose a significant health threat in the US. The range of tick vectors and burden of TBDs have been increasing over the last couple of decades, and improved diagnostics have facilitated the identification of new pathogens. Unfortunately, the diagnosis of TBDs can be challenging due to non-specific and overlapping symptoms, and a battery of tests is often required to adequately assess patients. Metagenomic next-generation sequencing is a promising method for the unbiased detection of existing and novel pathogens but is not widely recommended as a first-line test at this time and is unlikely to replace the need for other diagnostic methods, including serology, in the near future.
